# Assessment of antioxidant properties in selected pigmented and non-pigmented rice (*Oryza sativa* L.) germplasm and determination of its association with *Rc* gene haplotypes

**DOI:** 10.1186/s12870-024-05623-2

**Published:** 2024-09-28

**Authors:** Srikanthi Rebeira, Dimanthi Jayatilake, Rohitha Prasantha, Thamali Kariyawasam, Lalith Suriyagoda

**Affiliations:** 1 Postgraduate Institute of Agriculture, University of Peradeniya, Peradeniya, Sri Lanka; 2 Food Research Unit, Department of Agriculture, Peradeniya, Sri Lanka; 3 Department of Agricultural Biology, Faculty of Agriculture, University of Peradeniya, Peradeniya, Sri Lanka; 4 Department of Food Science and Technology, Faculty of Agriculture, University of Peradeniya, Peradeniya, Sri Lanka; 5 Department of Crop Science, Faculty of Agriculture, University of Peradeniya, Peradeniya, Sri Lanka

**Keywords:** Phenolics, Flavonoids, Proanthocyanidins, Radical scavenging, Molecular markers

## Abstract

**Background:**

Antioxidant properties of rice provide various health benefits due to its ability to inhibit cellular oxidation. Antioxidant content of rice is known to be linked with the pericarp pigmentation. The *Rc* gene of rice (*Os07g0211500*) codes for a basic helix-loop-helix (bHLH) protein, acting as a transcriptional factor in regulating proanthocyanidin biosynthesis. The current study was carried out to evaluate the variation of antioxidant properties in a selected panel of rice accessions and assess the possibility of using haplotypes defined based on the *Rc* gene to predict pericarp pigmentation and antioxidant content in rice.

**Results:**

Thirty-two rice accessions were evaluated for grain pericarp colour and antioxidant properties; total phenolic content (TPC), total flavonoids (TFC), proanthocyanidins (PAC) and radical scavenging activity (RSA). The parameters TPC, TFC and PAC showed significant positive correlation with RSA (*r* > 0.69; *P* < 0.01). The study panel showed a wide variation for antioxidant properties and rice accessions such as *Sudu Heenati*,* Deweraddiri*,* Madathawalu*, *Masuran*, Ld 368, At 311, *Kalu Heenati*, Bw 272-6B, *Pokkali*, At 362 and *Wanni Dahanala* exhibited profound potential with respect to antioxidant properties. Based on three-target sites previously reported as critical for the function of the coded bHLH protein (an A/C SNP at 1,353-bp, a 1-bp insertion/deletion at 1,388-bp, and a 14-bp insertion/deletion at 1,408-1,421-bp positioned in the mRNA corresponding to the exon 6 of rice *Rc* gene), three haplotypes were defined (H1-H3). Pigmentation of the rice pericarp could be successfully explained based on the defined haplotypes (H1 (C/G/+): red, and H2 (A/G/+) and H3 (C/G/-): white), and the H1 haplotype corresponded to a significantly (*P* < 0.05) higher TPC, TFC, PAC and RSA compared to the other haplotypes.

**Conclusions:**

The studied rice accessions showed a significant variation with respect to antioxidant properties. Haplotype H1 defined based on the three-target sites in the exon 6 of *Rc* gene can detect rice accessions with red pigmented pericarp and high antioxidant properties effectively. Hence, its use can be recommended as an alternative to biochemical assays for screening during rice breeding programs.

**Supplementary Information:**

The online version contains supplementary material available at 10.1186/s12870-024-05623-2.

## Background

Rice (*Oryza sativa* L.) is the staple food of many Asian countries and is considered to be the main source of nutrients and functional components such as dietary fiber and antioxidants [1, 2]. Rice grain with an intact bran layer is more nutritious and contains more dietary fat, fiber, minerals, vitamins, and health-promoting bioactive phytochemicals such as phenolics, flavonoids, γ-oryzanol, tocopherols, tocotrienols, ferulic and phytic acids, compared to fully milled white rice [1, 3]. These bioactive components carry potential health benefits against infectious and chronic diseases due to their ability to inhibit cellular oxidation through radical scavenging activity [1, 4, 5]. Many traditional rice accessions are presented with pigmented pericarps, coloured red, purple, black, brown, yellow, and green. The kernel colour differs due to various biochemical compounds such as phenolic acids, anthocyanins, and proanthocyanidins in the pericarp. The colour in red pigmented rice is due to either proanthocyanidins or anthocyanins present in the rice bran, which is responsible for its higher antioxidant capacity compared to other non-pigmented rice accessions [1, 2, 5]. Ability of pigmented rice to reduce the oxidative stress by suppressing reactive oxygen species and increasing the activity of antioxidant enzymes has been demonstrated by cell culture studies carried out by Chiang et al. [6]. Previous studies have shown that the use of antioxidants in pigmented rice may help to reduce type II diabetic risk [5, 7, 8]. Due to its richness in antioxidants, rice has a high potential to serve as a source for commercial production of antioxidants.

Rice germplasm of Sri Lanka consists of nearly 2,400 traditional accessions and more than 80 improved accessions of pigmented and non-pigmented rice [2, 9]. Most of these accessions remain uncharacterized and underutilized at large. Previous studies have reported a considerable variation in antioxidant properties and highlighted the antioxidant richness in rice accessions such as *Kalu Heenati*, *Sudu Heenati*, *Beheth Heenati*, *Pachchaperumal*, *Dik Wee*, At 362, At 353, and H 4 [1, 2, 8–10]. However, comprehensive studies have not been conducted for whole grain rice representing a genetically diverse panel consisting of both pigmented and non-pigmented Sri Lankan rice accessions. Lack of knowledge on antioxidant properties limits the commercial utilization of available rice accessions in various industries, therefore, such investigations are considered to be a timely requirement.

Antioxidant content of rice is known to be linked with the pericarp pigmentation and the degree of rice polishing [5, 7, 11]. Marker-assisted selection (MAS) using molecular markers targeting quantitative trait loci (QTL)/candidate genes associated with antioxidant properties is a promising method to complement selections done in rice improvement programs, targeting development of rice accessions with better antioxidants properties [12]. Given the labour, time and cost intensive screenings involved with the estimation of antioxidant properties based on chemical assays, a MAS approach will facilitate early screening of desired progeny in breeding programs with high precision and cost effectiveness. Shao et al. [13] reported significant marker-trait associations for phenolic content, flavonoids content and antioxidant capacity with markers linked to several major and minor QTL/genes.

In rice, pericarp pigmentation by anthocyanin and other compounds including proanthocyanidins are largely regulated by *Rc*, *Rd* and *Ra* genes [13–16]. The *Ra* gene is known to convey the purple/black pericarp due to anthocyanins, while *Rc* and *Rd* genes are responsible for red/brown pericarp due to proanthocyanidins [13, 14]. Following classical genetics *Rc/Rd* combination results a red pericarp, *Rc/rd* a brown pericarp and *rc/Rd* a white pericarp [15]. Hence, the *Rc* gene is a key regulator of pericarp colour in rice.

The *Rc* gene in *Oryza rufipogon* codes for a basic helix-loop-helix (bHLH) protein and carries a functionally important bHLH domain at the end of its exon 6 and at the beginning of the exon 7 [15]. The bHLH protein is a transcription factor involved in anthocyanin biosynthesis in rice [16]. A haplotype analysis revealed that a single frame-shift mutation event in the *Rc* gene (*Os07g0211500*) is mainly responsible for the occurrence of non-pigmented rice (with a white pericarp) from the pigmented wild rice ancestors (with a red pericarp) due to knocking off of proanthocyanidin production [17]. In rice accessions such as *Kasalath* and H75, the intact bHLH protein results in a pigmented red pericarp (*Rc* allele). However, in rice accessions such as *Nipponbare* a 14-bp deletion at 1,408–1,421-bp position on the mRNA (corresponding to exon 6 of *Rc* gene; hereinafter referred to as the 14-bp InDel) creates a frameshift mutation, resulting a stop codon and prematurely truncating the protein ahead of the bHLH domain, leading to a non-pigmented white pericarp (*rc* allele). This mutation originally occurred in a genetic background of *japonica* rice subspecies and was later introgressed to all other rice subpopulations in the world [17]. Further, a SNP at 1,353-bp position in the mRNA (corresponding to an Adenine/Cytosine SNP at exon 6 of *Rc*; hereinafter referred to as the A/C SNP) and a 1-bp InDel at 1,388-bp position in the mRNA (corresponding to a Guanine/- InDel at the exon 6 of *Rc*; hereinafter referred to as the 1-bp InDel) was previously reported as critical for its functionality [15, 17, 18]. Sweeny et al. [15], reported that an Adenine at the A/C SNP creates a premature stop codon ahead of the bHLH domain and thereby makes the 1-bp InDel and/or 14-bp InDel positioned downstream to it redundant [*Rc-s* allele; 17]. Hence, Sweeney et al. [15, 17] reported that this A/C SNP mutation makes rice grains with white or light red pigmented pericarps, despite having a sequence present at the 14-bp InDel. Further, a naturally occurring rare 1-bp InDel mutation closer to the 14-bp InDel was reported by Brooks et al. [18]. There the deletion of 1-bp results a frameshift in the amino acid sequence, restoring the impact of the 14-bp deletion and eventually producing a pigmented rice [*Rc-g*; 18]. Therefore, for an accurate prediction of the pericarp colour it is important to focus on these critical sites together as one haplotype rather than individually.

Based on the polymorphisms found on the entire *Rc* gene in a diverse panel of rice, Sweeney et al. [17] defined 11 haplotypes for *Rc* gene. In the current study, considering the involvement of *Rc*-bHLH protein as a transcriptional factor in proanthocyanidin biosynthesis, we propose to evaluate the feasibility of using an *Rc* gene haplotype defined based on three-critical sites corresponding to the exon 6 of *Rc* gene (A/C SNP, 1-bp InDel and 14-bp InDel) to predict the antioxidant content in rice.

## Materials and methods

### Sample preparation

The thirty-two rice accessions were selected to represent both pigmented and non-pigmented rice, by including 12 newly improved Sri Lankan accessions, 18 traditional Sri Lankan accessions and two traditional Indian accessions (Suplimentary Material 1), considering their popularity as cultivated rice accessions, and/or breeding potential. Seeds of these rice accessions were obtained from Rice Research and Development Institute, Bathalagoda, Sri Lanka. Seed samples were collected at physiological maturity, air-dried (31 ± 2 °C and 70 ± 2% relative humidity) to approximately 14% moisture content (wet basis) and were stored for 3 months at ambient conditions before further analysis. Samples were dehusked using a laboratory dehuller (THU 358, Satake, Japan) and flour samples of brown rice were prepared using Cyclotec mill (Foss Tecator 1093, Sweden), passing through a 0.1 mm sieve. Flour samples were stored in sealed polypropylene packages at -4 °C until further analysis.

### Determination of pericarp colour

To unbiasedly determine the pericarp pigmentation colour of rice accessions, approximately 50 g of brown rice grain sample was placed in a tray and L*, a* and b* values were taken as average of ten colourimeter readings (Colourimeter CS-10, China) randomly taken from each rice sample. The categorization as pigmented and non-pigmented clusters was done based on the range of L*, a* and b* values.

### Determination of antioxidant properties

Total antioxidants in the rice accessions were extracted according to the methods described by Butsat and Siriamornpun [4]. The total antioxidants in one gram of rice flour was extracted using 10 mL methanol (80% v/v) in a shaking water bath (BW200, Yamto, Japan) for 16 h at 150 rpm at room temperature. The extract was transferred to 50 mL centrifuge tubes and the supernatant was collected after centrifugation at 2,500 rpm for 20 min (Hitachi Himac CT 6D, Japan). The solvent was removed at 40 °C using a vacuum rotary evaporator (BUCHI Rotavapor R 200, Switzerland), and freeze-dried (Alpha 1–2 LD plus, Germany) extracts were stored in -20 ^o^C for future analysis. For the analysis of total phenolic content (TPC), total flavonoid content (TFC), proanthocyanidin content (PAC) and radical scavenging activity (RSA), the stored extracts were dissolved in methanol (100 µg/ mL) and all assays were performed in triplicates.

The TPC was determined using the methods described by Abeysekera et al. [9] and Butsat and Siriamornpun [4] with modifications, where 200 µL of previously prepared rice extract was mixed with 1,100 µL of freshly prepared 10 times diluted folin-ciocalteu reagent, followed by 700 µL of sodium carbonate solution (10% v/v). The after sample was incubated for 30 min at room temperature and the absorbance was measured at 765 nm using a UV-visible spectrophotometer (Optizen POP, Macasays, Korea). Using a standard concentration series of Gallic acid, TPC was calculated and expressed as mg Gallic acid equivalent (GAE) per 100 g of brown rice (dry weight basis).

The TFC was determined according to the methods described by Shen et al. [7]. In brief, 500 µL of rice extract was mixed with 2 mL of double distilled water, 150 µL of 5% sodium nitrite solution and 150 µL of 10% aluminum chloride (AlCl_3_.6H_2_O). The solution was allowed to stand for another 5 min and 1 mL of 1 M sodium hydroxide was added. The solution was kept standing for another 15 min and absorbance was measured at 415 nm using a UV-visible spectrophotometer. The TFC was calculated using a standard concentration series of Rutin and was expressed as mg Rutin equivalents (RE) per 100 g brown rice (dry basis).

The PAC was determined using vanillin-hydrochloric assay according to the methods described by Gunaratne et al. [1] with some modifications. In brief, the sample was prepared by mixing 400 µL of rice extract with 1 mL of sulfuric acid/ methanol solution and 1 mL of 1% vanillin in methanol (w/v). A control sample was prepared by adding 100% methanol instead of vanillin into methanol to eliminate the influence of non-vanillin reactive compounds. Sample and the control were incubated for 15 min at 30 °C and absorbance was measured at 500 nm using a UV-visible spectrophotometer. Reagent blanks for sample mixture and control mixture were prepared using methanol instead of the rice extract. The difference of absorbance in the sample and control mixtures against their reagent blanks was calculated using the following Eq. (1) and the corrected absorbance value was used to determine the PAC. A standard curve was developed using the absorbance values of a catechin standard and PAC was expressed as mg catechin equivalents (CTE) per 100 g brown rice (dry basis).

1 $$A = \left( {As - Ab} \right) - \left( {Ac - Aa} \right)$$

where, A was the corrected absorbance (at 500 nm); A_s_ and A_c_ were the absorbance (at 500 nm) of sample and control mixtures, respectively.

The RSA was determined according to the methods described by Abeysekera et al. [9]. A total of 50 µL of rice extract was mixed with 1,550 µL of methanol and 400 µL of 2,2-diphenyl1-picrylhydrazyl (DPPH) solution (20 mg DPPH in 100 mL of methanol). The mixture was incubated for 15 min in the dark at room temperature. The absorbance was measured with a UV-visible spectrophotometer at 515 nm against a methanol blank solution. The percentage of RSA was estimated by using the following Eq. (2).

2 $$\begin{gathered} RSA\,\left( \% \right) = \hfill \\\frac{{ABS\,of\,control\,at\,515\,nm - ABS\,of\,rice\,sample\,at\,515\,nm}}{{ABS\,of\,control\,at\,515\,nm}} \times 100 \hfill \\ \end{gathered}$$

where, ABS is absorbance at 515 nm wavelength.

## Defining exon 6 based ***Rc*gene haplotypes**

Genomic DNA was extracted from three-week old tender rice leaves using a modified CTAB method described by Doyle and Doyle [19] and was normalized to 50 ng/µL. The primer pairs *Rc_F/R* [13] amplifying the genomic region spanning over a 14-bp deletion in Rc gene (*Rc_F*: 5’-ATCAGTCCAGGCACCACA-3’ and *Rc_R*: 5’- CCAAAGATCGCAGAATTATGA-3’) was amplified in the study panel of 32 rice accessions. The PCR amplification was carried out in a thermal cycler (CT1000, Bio-Rad, USA) using a 15 µL final PCR volume containing 50 ng/µL of genomic DNA, 1× GoTaq Green master mix^®^, 0.66 µM of each primer and 1 mg/mL Bovine Serum Albumin. The PCR program consisted of a pre-denaturation at 95 °C for 5 min followed by denaturation at 95 °C for 30 s, annealing at a 52 °C for 30 s, 72 °C for 1 min for 35 cycles and with a final extension of 72 °C for 5 min. The PCR products were resolved in 3% Agarose pre-stained with 5% (v/v) ethidium bromide and was visualized using a UVC1-1100 UV gel documentation system (Major Science, USA). The PCR products were subjected to bi-directional Sanger sequencing at GeneLabs (Pvt.) Ltd. using SeqStudio Genetic Analyser (Thermo Fisher Scientific Inc., USA). The forward and reverse sequence chromatograms were visualized in Geneious V7.1.3 (Biomatters Inc, New Zealand) and were quality curated by trimming the end sequences and obtaining the consensus sequence. The sequence was subsequently trimmed to a 129-bp region spanning over the three-key target sites in the *Rc* gene (*Os07g0211500*); A/C SNP at position 1,353 bp, 1-bp InDel at position 1,388 bp and 14-bp InDel at position 1,408–1,421 bp in the mRNA.

The gene sequence of the *Rc* gene (*Os07g0211500*; chr07:6062889..6069317 ) of the *japonica* genome reference *Nipponbare* was retrieved from the Rice Annotation Project Database (RAP-DB; https://rapdb.dna.affrc.go.jp/index.html) and the gene features were annotated using Geneious V7.1.3. In addition, the *Rc* genomic sequence of *Kasanath* (AB247503) and H75 (DQ204735) representing red pericarp (*Rc*), *Jefferson* (DQ204736) representing white pericarp (*rc*), and *Surjamkuhi* (DQ204738) representing light red (*Rc-s*) was retrieved from NCBI (https://www.ncbi.nlm.nih.gov/). The trimmed 129-bp sequences of exon 6 of *Rc* gene amplified from the 32 rice accessions in the study panel (GenBank accession numbers: PP592897 - PP592928) spanning over the three-target sites, was aligned to the retrieved reference *Rc* gene sequences of *Nipponbare*, *Kasalath*, H75, *Jefferson* and *Surjamkuhi* in Geneious V7.1.3 using multiple alignment feature ClustalW. The sequence polymorphisms at the three-target sites (A/C SNP at position 1,353 bp, 1-bp InDel at position 1,388 bp and 14-bp InDel at position 1,408–1,421 bp corresponding to the mRNA) were scored and exon 6 based *Rc* haplotypes were identified based on variant pattern.

### Statistical analysis

Data TFC, TPC, PAC and RSA were tested for normality using Shapiro-Wilk Normality Test (*P* = 0.05) and were transformed using rank-based inverse normal transformation to achieve normality using a Phython code executed in Google Collaboratory. One-way ANOVA was performed and mean comparisons were conducted using Tukey HSD method at *P* < 0.05 in Minitab V17 (Minitab LLC., USA). Non-parametric Spearman rank correlation coefficient techniques were used to determining the strength of the relationship among TFC, TPC, PAC, RSA and Lab color values L*, a* and b* at *P* < 0.05 in SPSS V22 (IBM Corp., USA). Antioxidant properties of each rice accession was visualized using Orange software V3.36.2 [20]. A hierarchical cluster analysis was performed based on the euclidean distance (average linkage) method to group the rice accessions according to their antioxidant properties in Minitab V17. The association of the traits TPC, TFC, PAC and RSA with the defined haplotypes were tested using one-way ANOVA and mean comparison was conducted using Tukey HSD method in Minitab V17.

## Results

**Fig. 1:**
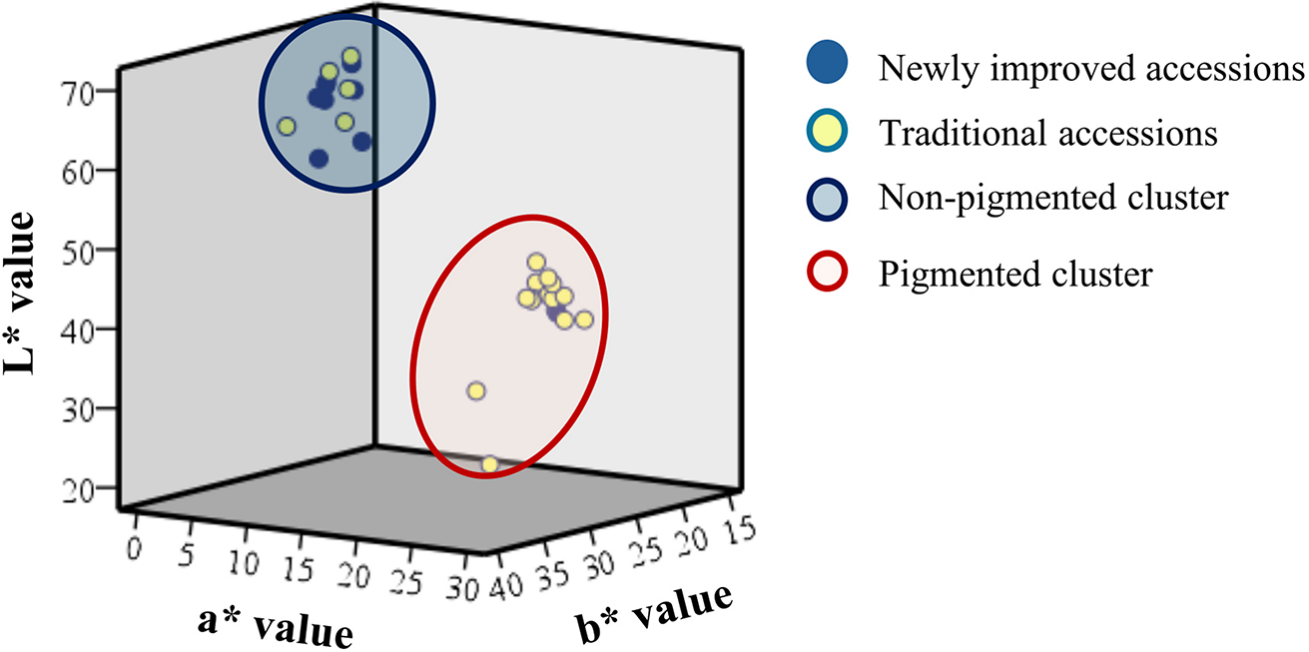
Clustering of pigmented and non-pigmented rice accessions according to L*, a* and b* values

The rice accessions in the study panel can be clustered into two distinct groups based on L*, a* and b* values. The L* value from lower to higher value (0–100) represents darkness to whiteness of the brown rice samples. Among the pigmented accessions, L* value ranged from 26.22 ± 1.16 to 46.18 ± 2.84 in *Dik Wee* and *Masuran*, respectively, while among the non-pigmented accessions it ranged from 56.65 ± 0.86 to 69.41 ± 1.15 in At 306 and *Rathdel*, respectively. The a* values that represent greenness to redness (negative to positive) was higher in pigmented accessions ranging between 14.69 ± 1.67 to 27.50 ± 3.21 in *Sulai* to *Dik Wee* compared to non-pigmented accessions Bg 358 and *Suduru samba*, ranging between 0.55 ± 0.21 to 2.84 ± 0.43, respectively. The b* values that represent blueness to yellowness in pigmented rice accessions ranged from 15.09 ± 1.06 to 35.90 ± 4.38 in *Hondarawalu* and *Dik Wee*, respectively, and in non-pigmented rice b* values ranged between 18.67 ± 1.34 to 26.88 ± 1.37 in Bg 94 − 1 to *Suwandel*, respectively. Hence, based on the range of L* and a* values pigmented and non-pigmented rice accessions could be categorized into two distinct clusters, however, b* values did not contribute to this distinctness (Fig. 1). In the pigmented rice, the majority was traditional accessions (15 out of 19), and in the non-pigmented rice the majority was improved accessions (8 out of 13).

### Antioxidant properties of pigmented and non-pigmented rice

The TPC among the studied rice accessions ranged from 27.32 ± 0.40–282.23 ± 1.82 mg GAE/100 g (Table 1). According to Table 1, TPC of pigmented rice accessions varied considerably compared to TPC of non-pigmented rice accessions (CV of pigmented: 35.3% and non-pigmented: 26.1%). The majority of the pigmented rice showed a significant difference (*P* < 0.05) in the average TPC when compared to non-pigmented rice. The highest TPC among the pigmented rice accessions were in *Sudu Heenati* and *Madathawalu* (282.23 ± 1.82 and 279.8 ± 4.3 mg GAE/100 g) and their TPC was significantly higher (*P* < 0.05) compared to other rice accessions. Similarly, in non-pigmented accessions the highest TPC was reported in At 405 (73.36 ± 1.48 mg GAE/100 g), which was not significantly different (*P* > 0.05) to non-pigmented rice accessions Bg 360, *Gonabaru*, Bg 94 − 1, Bg 366 and Bg 358 and pigmented accessions *Dular*, *Pachchaperumal*, *Hondarawalu*, At 362, *Kuruluthuda* and *Herathbanda*.

**Table 1:** Variation of antioxidant properties of selected pigmented and non-pigmented rice accessions

Accession	Total phenolic content (mg GAE/100 g)*	Total flavonoid content (mg RE/100 g)	Proanthocyanidin content (mg CTE/100 g)	DPPH radical scavenging activity (%)
**Pigmented rice accessions**				
*Sudu Heenati*	282.23 ± 1.82^a^	38.07 ± 0.65^jklm^	165.05 ± 1.11^fghij^	91.35 ± 0.70^a^
*Madathawalu*	279.80 ± 4.30^a^	92.02 ± 0.58^bc^	192.18 ± 1.11^cdef^	90.74 ± 0.45^abc^
*Kahata wee*	243.07 ± 7.43^b^	65.31 ± 0.57^fgh^	241.93 ± 4.83^bc^	86.49 ± 1.99^bcdefghi^
*Sulai*	185.10 ± 3.11^bcd^	34.11 ± 0.92^klmno^	155.10 ± 2.93^ghijk^	83.93 ± 0.45^cdefghij^
*Deveraddiri*	173.24 ± 6.10^bcde^	95.72 ± 0.18^b^	179.52 ± 2.93^efghi^	90.93 ± 1.41^a^
*Kalu Heenati*	167.39 ± 0.96^cdef^	65.66 ± 0.37^fgh^	185.85 ± 1.92^defgh^	91.36 ± 0.87^ab^
*Pokkali*	164.23 ± 2.10^defg^	57.68 ± 0.60^ghi^	203.04 ± 5.54^bcd^	88.74 ± 1.09^abcdef^
*Dik wee*	157.34 ± 6.19^efgh^	70.64 ± 1.38^def^	154.20 ± 2.93^ghijk^	79.69 ± 1.19^defghijk^
*Masuran*	151.82 ± 3.62^fghij^	97.70 ± 1.25^a^	198.51 ± 1.11^cde^	89.45 ± 0.33^abcd^
*Wanni Dahanala*	131.33 ± 0.79^ghijk^	80.20 ± 1.04^ed^	115.31 ± 1.92^ijklmn^	85.22 ± 3.75^abcdefgh^
*Dular*	126.65 ± 1.22^hijkl^	36.27 ± 1.08^klmn^	193.99 ± 11.98^cdef^	82.61 ± 2.90^defghijk^
*Pachchaperumal*	123.23 ± 1.48^hijkl^	38.98 ± 0.88^ijkl^	202.13 ± 5.75^bcd^	85.07 ± 1.07^cdefghij^
*Hondarawalu*	104.98 ± 0.80^ijklm^	67.47 ± 0.77^ef^	188.57 ± 1.92^defg^	86.50 ± 2.43^bcdefghi^
*Kuruluthuda*	96.83 ± 1.36^klmn^	65.69 ± 0.89^fg^	145.16 ± 1.92^hijkl^	54.43 ± 3.30^defghijk^
*Herathbanda*	77.91 ± 1.02^lmno^	72.74 ± 0.84^cde^	164.15 ± 5.07^fghij^	82.89 ± 1.70^defghij^
Bw 272/6B	235.31 ± 3.16^bc^	38.90 ± 1.00^ijkl^	188.57 ± 1.92^defg^	86.84 ± 3.36^abcdef^
Ld 368	161.26 ± 0.81^efgh^	28.60 ± 1.36^mnop^	333.27 ± 3.99^a^	89.37 ± 0.56^abcde^
At 311	160.69 ± 0.81^efghi^	58.98 ± 1.84^ghi^	306.14 ± 2.21^ab^	89.63 ± 0.36^abcd^
At 362	102.57 ± 0.49^jklmn^	39.01 ± 0.53^ijk^	175.91 ± 2.93^fghi^	88.77 ± 0.61^abcdefg^
Average^#^	164.47 ± 3.11	60.20 ± 1.15	194.14 ± 2.67	85.47 ± 0.43
CV (%)	35.33	35.67	26.78	9.77
**Non-pigmented rice accessions**				
*Gonabaru*	56.25 ± 0.20^nop^	19.24 ± 0.89^pqr^	85.47 ± 3.32^jklmno^	31.96 ± 0.06^hijklm^
*Suduru Samba*	47.20 ± 0.84^pqr^	24.11 ± 0.57^nopq^	129.78 ± 6.17^ijklm^	20.98 ± 2.26^lmn^
*Inginimitiya*	36.10 ± 1.42st	18.26 ± 0.64^pqrs^	88.18 ± 1.92^jklmno^	10.57 ± 0.09^n^
*Rathdal*	31.89 ± 0.66^t^	32.52 ± 0.74^lmno^	75.06 ± 0.55^mnopq^	16.57 ± 2.70^mn^
*Suwandel*	27.32 ± 0.40^u^	5.13 ± 0.45^w^	81.85 ± 2.93^klmno^	21.05 ± 2.30^lmn^
At 405	73.36 ± 1.48^lmno^	44.59 ± 0.67^hij^	65.57 ± 2.21^rs^	31.91 ± 2.90^ijklm^
Bg 360	64.60 ± 0.45^mno^	13.40 ± 0.43st	66.93 ± 3.63^qrs^	27.08 ± 1.33^klm^
Bg 94 − 1	51.78 ± 2.44^opq^	20.87 ± 0.71^opq^	73.71 ± 4.43^nopqr^	45.10 ± 1.47^efghijkl^
Bg 366	50.37 ± 0.18^opq^	10.55 ± 0.32^tu^	73.26 ± 2.54^opqr^	30.41 ± 0.52^jklm^
Bg 358	50.20 ± 0.82^opq^	17.58 ± 0.61^qrs^	72.80 ± 1.11^opqr^	29.87 ± 1.20^jklm^
Bg 352	45.00 ± 2.02^qrs^	9.23 ± 0.28^uv^	78.23 ± 2.93^lmnop^	36.69 ± 1.76^ghijklm^
At 306	44.00 ± 1.19^qrs^	15.47 ± 0.94^rst^	57.43 ± 3.99^s^	43.40 ± 5.85^fghijkl^
Bw 267-3	40.86 ± 3.63^rst^	8.78 ± 0.48^v^	69.19 ± 3.32^pqr^	34.92 ± 3.48^hijklm^
Average^#^	47.61 ± 0.97	18.44 ± 0.82	78.27 ± 1.44	29.27 ± 0.77
CV (%)	26.10	56.61	22.42	34.79

*Values were expressed as mean ± SE (*n* = 3). Mean values in each column superscripted by different simple letters are significantly different at *P* < 0.05. ^#^Mean ± SE for each category (pigmented/ non-pigmented)

According to the results of the present study (Table 1), TFC ranged from 97.70 ± 1.26–5.13 ± 0.45 mg RE/100 g, with highest and the lowest TFC recorded in the pigmented rice *Masuran* and non-pigmented rice *Suwandel*, respectively. Most pigmented rice showed significantly higher (*P* < 0.05) mean TFC compared to the non-pigmented rice. Among the non-pigmented rice accessions At 405 (44.59 ± 0.67 mg RE/100 g) showed a significantly higher (*P* < 0.05) TFC compared to all other non-pigmented rice accessions and some of the pigmented rice accessions such as *Dular*, *Sulai* and Ld 368. The TFC of *Masuran* was the highest in the pigmented category, showing a two-fold higher TFC than At 405, representing the highest TFC in the non-pigmented category (Table 1).

The PAC of the studied rice accessions ranged between 57.43 ± 4.00–333.27 ± 4.00 mg CTE/100 g (Table 1) for Ld 368 and At 306, respectively. It is noteworthy to report that the accession At 311 (306.14 ± 2.21 mg CTE/100 g) showed no significant difference (*P* > 0.05) to Ld 368 in terms of PAC. Comparison between pigmented and non-pigmented rice accessions showed that the pigmented rice accessions have a broader PAC range (115.31 ± 2.00–333.27 ± 4.00 mg CTE/100 g) compared to the non-pigmented rice accessions (57.43 ± 4.00–129.78 ± 6.20 mg CTE/100 g), and most of the pigmented rice accessions have a significantly higher (*P* < 0.05) PAC compared to non-pigmented rice accessions.

The RSA of phenolic fractions of the evaluated rice accessions ranged from 10.57 ± 0.10–91.36 ± 0.87% in *Inginimitiya* and *Sudu Heenati*, respectively (Table 1). The rice accessions *Deweraddiri*, *Kalu Heenati*, *Madathawalu*, At 311, *Masuran*, Ld 368, Bw 272-6B, *Pokkali*, At 362, and *Wanni Dahanala* was not significantly different (*P* > 0.05) to *Sudu Heenati* and formed the group exhibiting the highest RSA. With respect to RSA of non-pigmented rice, the highest value was reported by the rice accession Bg 94 − 1 (45.10 ± 1.47%), however, the non-pigmented rice accessions At 306, Bg 352, Bw 267-3, *Gonabaru*, At 405, Bg 366, Bg 358, Bg 360, *Suduru Samba*, and *Suwandel* were not significantly different (*P* > 0.05) to Bg 94 − 1 (Table 1). The pigmented rice accessions show lower variation compared to non-pigmented accessions (CV of pigmented rice − 9.8% and CV of non-pigmented rice − 34.8%), however, the range of RSA in pigmented rice accessions (54.43 ± 3.30–91.35 ± 0.70%) was broader compared to the non-pigmented rice accessions (10.57 ± 0.09–45.10 ± 1.47) due to the RSA values reported for the pigmented rice accession *Kuruluthuda* (54.43 ± 3.30%).

**Fig. 2:**
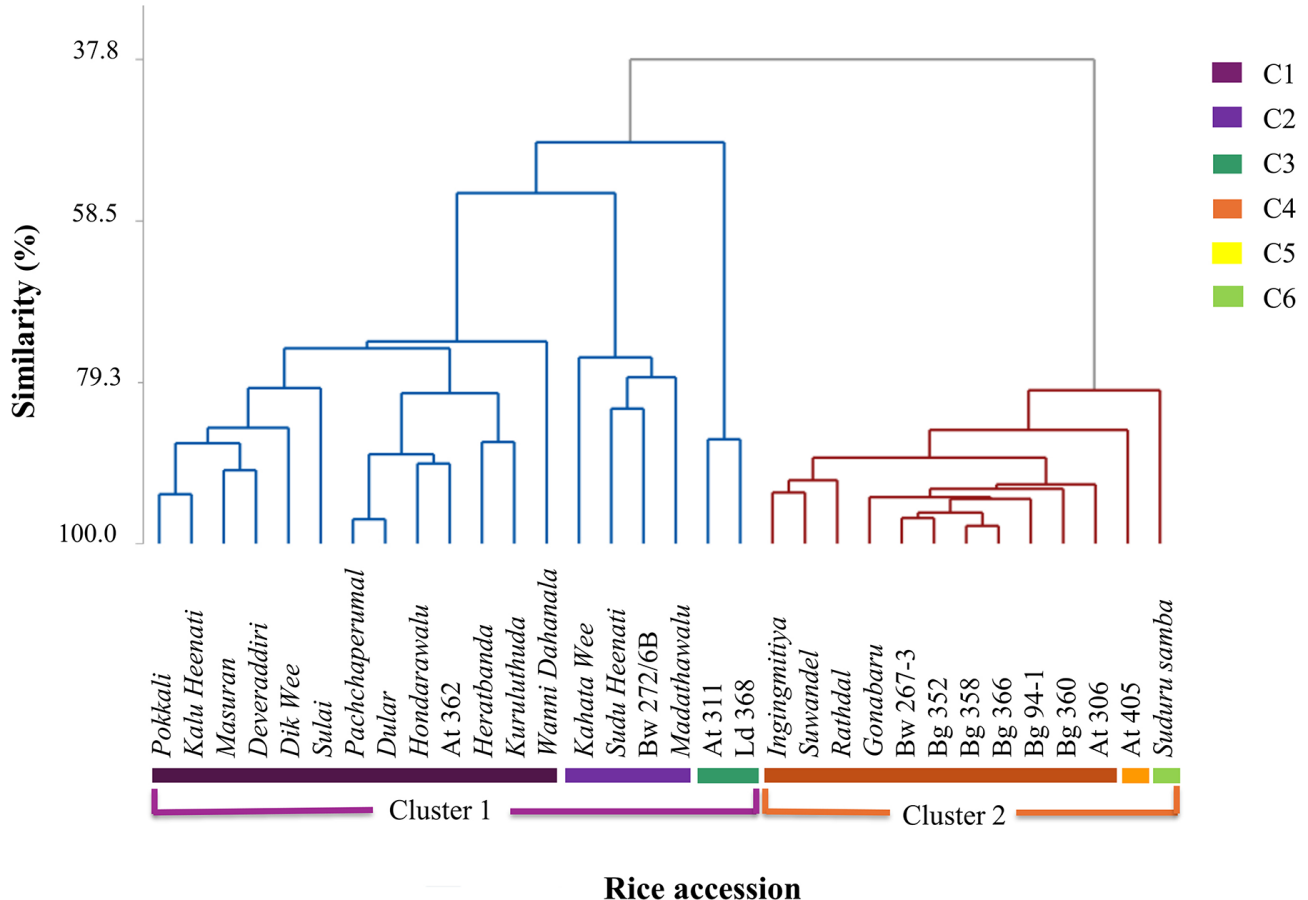
Dendrogram clustering of selected pigmented and non-pigmented rice accessions based on antioxidant properties. The sub-clusters are identified as C1-C6

Dendrogram in Fig. 2 reflects the clustering of the rice accessions in the study panel based on the tested antioxidant properties TPC, TFC, PAC, RSA and their pericarp colour. At a similarity of 37.8% two distinct clusters were identified corresponding to the pericarp colour; pigmented Cluster 1 and non-pigmented Cluster 2 (Fig. 2). The Cluster 1 could be sub-clustered to three groups at a similarity of 55.0% (C1-C3) and Cluster 2 can be further sub-clustered into three groups at a similarity of 85.4% (C4-C6). These sub-clusters revealed specific properties with respect to antioxidant components TPC, TFC and PAC. An important observation on the rice accessions grouped into the Cluster 1 sub-cluster C2 (*Kahata Wee*, *Madathawalu*, *Sudu Heenati*, Bw 272-6B) was the capturing of the highest TPC among the pigmented rice accessions. Further, in the Cluster 1 sub-cluster C3 (At 311 and Ld 368), the highest PAC among all the investigated pigmented rice accessions were captured. In the Cluster 2, the two rice accessions At 405 (sub-cluster C5) represented the highest TFC and TPC, and *Suduru Samba* (sub-cluster C6) the highest PAC among the non-pigmented rice accessions.

**Fig. 3:**
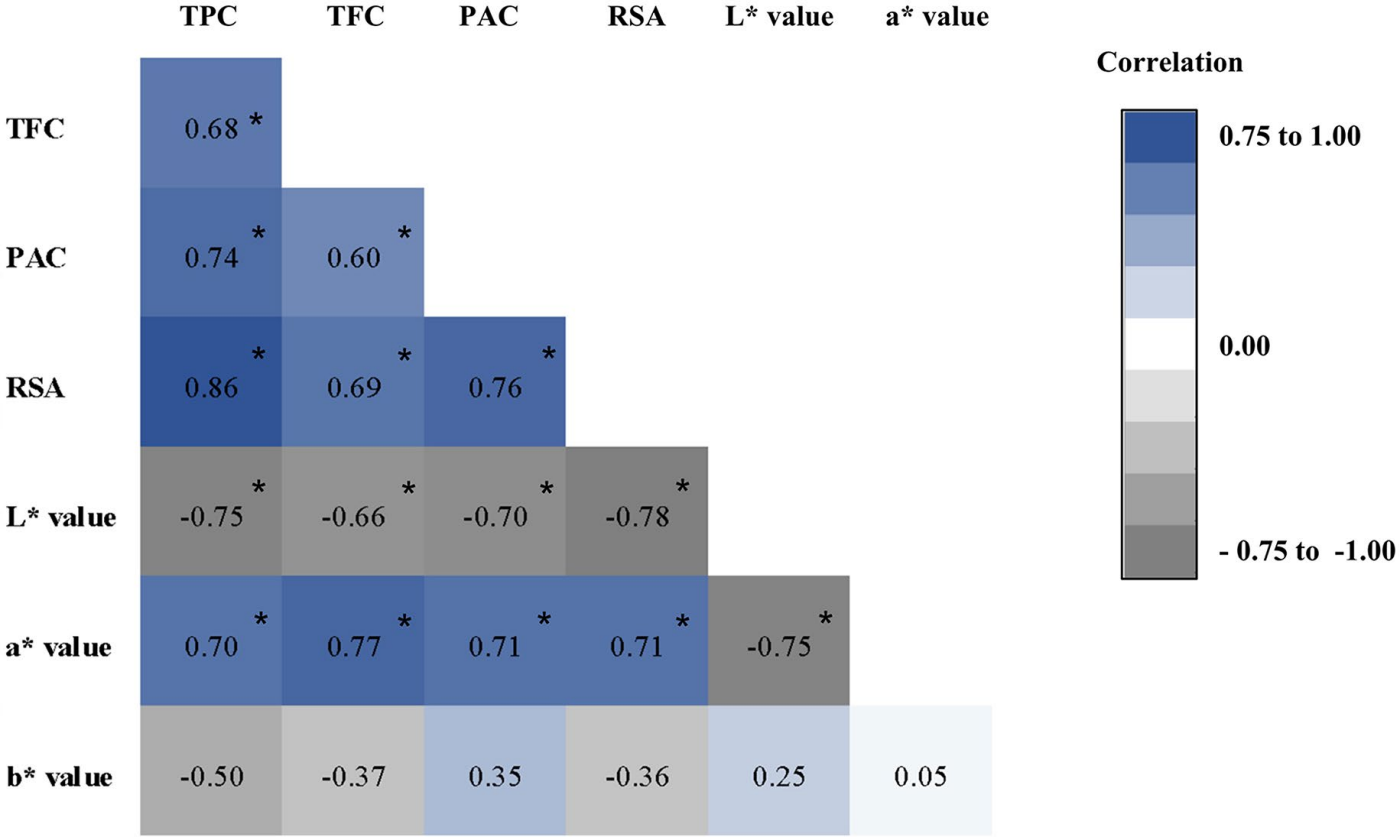
Spearman association rank correlation coefficient among antioxidant properties. Significant correlations (*P* < 0.01) among the total phenolic content (TPC), total flavonoid content (TFC), proanthocyanidin content (PAC), radical scavenging activity (RSA) and colourimeter readings; L*, a* and b* values are represented by an asterix (*)

As shown in Fig. 3, all the antioxidant parameters (TPC, TFC, PAC, and RSA) exhibited a significantly higher degree of association in Spearman rank correlations (*r* > 0.60, *P* < 0.01). The highest Spearman rank correlation among antioxidant properties was observed between TPC and RSA (*r* = 0.86, *P* < 0.01), while the lowest correlation was reported between TFC and PAC (*r* = 0.60, *P* < 0.01). The RSA showed a significant positive correlation with TFC (*r* = 0.69, *p* < 0.01) and PAC (*r* = 0.76, *p* < 0.01).

**Fig. 4:**
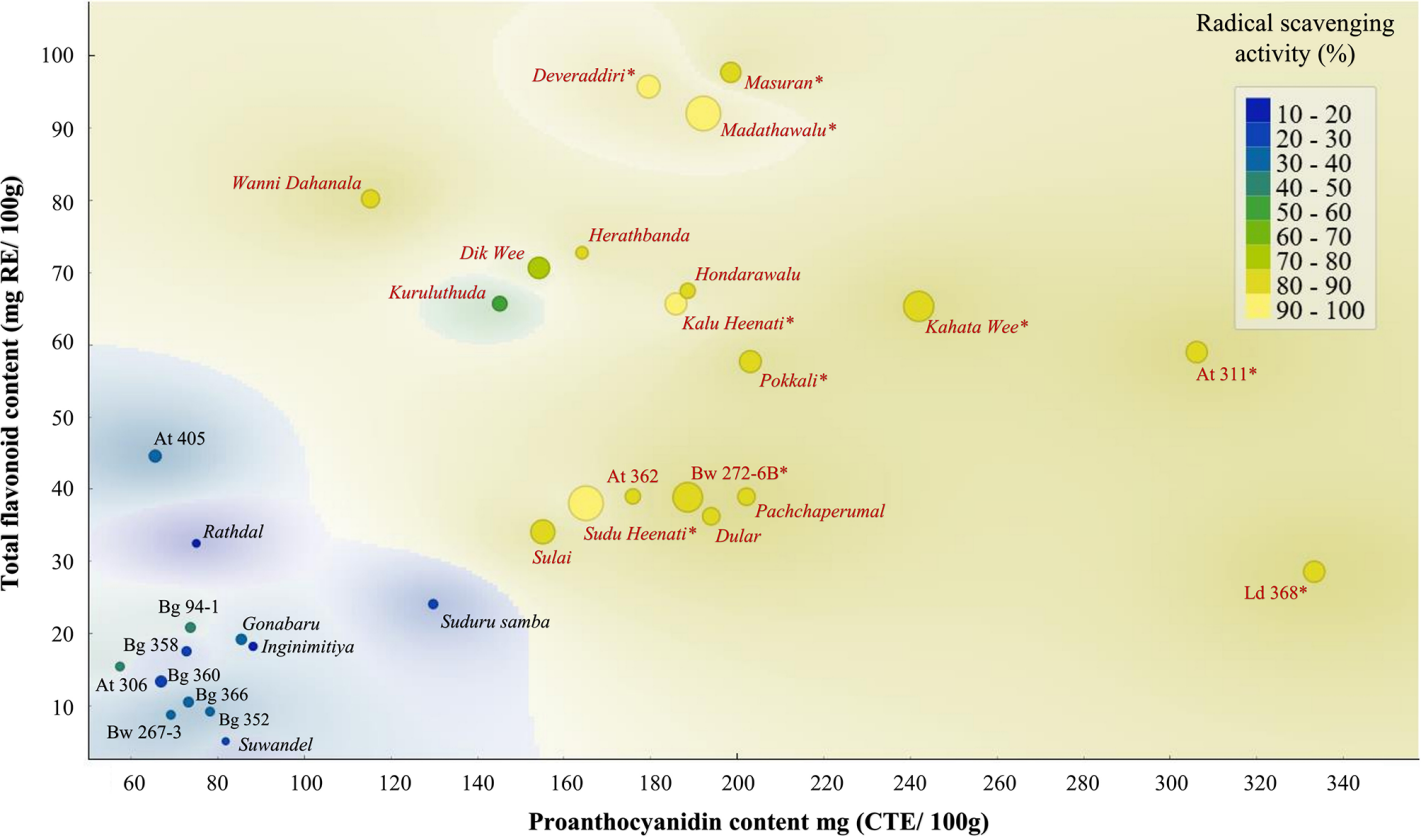
Antioxidant properties of pigmented and non-pigmented rice accessions. The text colour maroon indicates pigmented rice accessions and black non-pigmented rice accessions. The colour scale blue to light yellow indicates increasing trend of radical scavenging activity and size of the circles from smaller to larger indicates increasing trend of total phenolic content. The asterisk (*) indicates the rice accessions with highest values considering total phenolic content, total flavonoid content, proanthocyanidin content and radical scavenging activity

The Fig. 4 illustrates the antioxidant properties TPC, TFC, PAC and RSA of the 32 rice accessions, where the pigmented and non-pigmented accessions were clearly placed in distinct clusters. According to Fig. 4, the pigmented accessions; *Kahata Wee*, *Madathawalu*, *Deweraddiri*, *Masuran*, *Kalu Heenati*, *Sudu Heenati*, *Pokkali*, At 311, Bw 272-6B and Ld 368 have shown higher values with respect to all antioxidant parameters. Based on their high PAC and TFC, these accessions were placed in the upper limits of the plotted graph and was further demarcated by light yellow colour and larger circles based on their high RSA and TPC, respectively.

## Defining exon 6 based *Rc* gene haplotypes and deducing its correlation with antioxidant properties

**Fig. 5:**
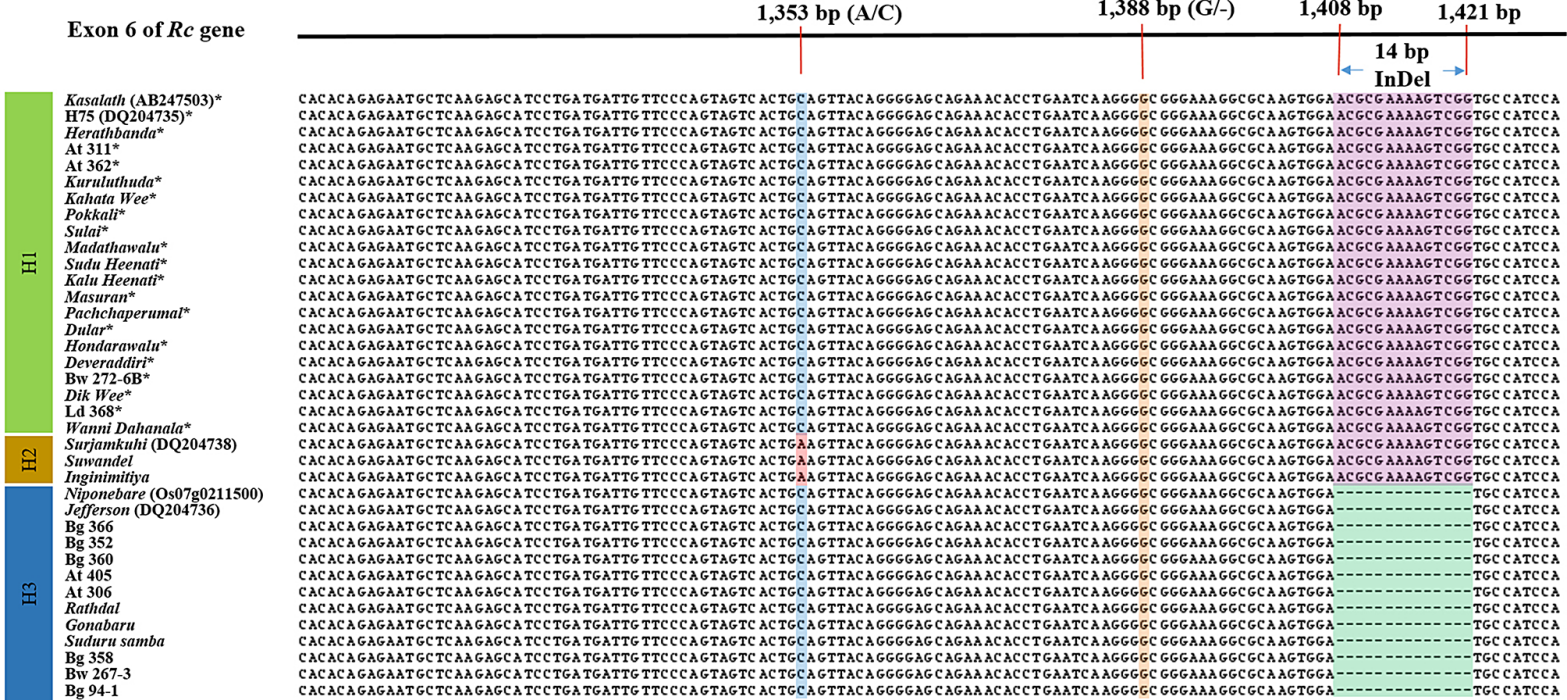
Graphical illustration of haplotypes (H1-H3) defined based on exon 6 polymorphisms of *Rc* gene in rice. The figure includes sequences from 32 rice accessions and reference sequences from red pericarp *Kasalath* (AB247503) and H75 (DQ 204735), light red pericarp *Surjamkuhi* (DQ 204738) and white pericarp *Jefferson* (DQ 204736) and *Nipponbare* (*Os07g0211500*). Pigmented rice accessions are indicated by asterisks (*)

Sequencing and aligning fragments amplified from the *Rc_F/R* primer pair [sized 215-bp and 229-bp; 13] confirmed the presence of a 14-bp deletion of *Rc* gene in 11 non-pigmented rice accessions, and the presence of sequence in the said position in 19 pigmented and two non-pigmented rice accessions, *Inginimitiya* and *Suwandel* (Fig. 5; 1,408-1,421 bp position). Hence, the 14-bp InDel alone could not accurately differentiate pigmented rice from non-pigmented rice. At the A/C SNP site located proximal to the 14-bp InDel, the accession *Inginimitiya* and *Suwandel* revealed an Adenine as opposed to the C reported by the rest of the rice accessions in the panel (Fig. 5: 1,353 bp position). With respect to the 1-bp InDel reported proximal to the 14-bp InDel and distal to the A/C SNP (Figs. 1 and 5,388 bp position), all accessions in the study panel revealed the presence of a Guanine and the rare 1-bp deletion could not be detected.

All the antioxidant properties including mean TPC, TFC, PAC and RSA of the H1 haplotype representing the pigmented rice accessions was significantly (*P* < 0.05) higher compared to that of H2 and H3 haplotypes representing the non-pigmented rice accessions. With respect to H2 and H3 haplotypes, though they did not differ significantly (*P* > 0.05) for mean of TPC, TFC and PAC, the mean of RSA in H2 haplotype was significantly higher (*P* < 0.05) compared to that of H3 haplotype.

**Table 2:** Antioxidant properties of exon 6 based *rc* gene haplotypes

Haplotype	Total phenolic content (mg GAE/100 g)*	Total flavonoid content (mg RE/100 g)*	Proanthocyanidin content (mg CTE/100 g)*	DPPH radical scavenging activity (%)*
H1	164.47 ± 58.11^a^	60.20 ± 21.48^a^	194.14 ± 2.67^a^	85.47 ± 8.35^a^
H2	31.71 ± 4.99^b^	11.69 ± 7.23^b^	85.01 ± 0.05^b^	15.81 ± 6.10^c^
H3	50.50 ± 11.11^b^	19.67 ± 10.54^b^	77.04 ± 0.19^b^	31.72 ± 8.79^b^

*Values were expressed as mean ± SD. Mean values in each column superscripted by different simple letters are significantly different at *P* < 0.05

## Discussion

Anthocyanins and proanthocyanidins are known to be the main pigments responsible for pericarp colour in rice [1, 5]. Antioxidants in rice are associated with pericarp pigmentation and higher phenolic content. These phenolic components are known to have potential health benefits and are used to treat human diseases, including cancer, cardiovascular diseases and inflammatory diseases [5, 7, 11]. In the study panel, the majority of pigmented rice were traditional accessions, and the majority of the non-pigmented rice was improved accessions. Comparable results have been reported in previous studies [9, 21, 22] indicating that majority of the Sri Lankan traditional rice accessions have red pigmented pericarps except for few white pericarped accessions such as *Suwandel*, *Gonabaru*, *Suduru Samba*, *Inginimitiya*, and *Rathal.* Similarly, agreeing with previous studies [9, 21, 22], most of the improved Sri Lankan rice accessions in the study panel carried white pericarps.

Phenolic compounds including phenolic acids, flavonoids and tannins are the main constituents that show antioxidant activity in rice [13, 23]. The TPC of pigmented rice accessions reported higher values and varied considerably compared to the values reported by non-pigmented rice accessions. The findings are comparable with previous studies, where TPC ranged between 117-388 mg GAE/100 g [5, 24]. However, compared to the values reported in the current study, lower TPC was reported by Gunaratne et al. [1] for the traditional pigmented accessions *Sudu Heenati*, *Kalu Heenati*, *Madathawalu*, *Sulai*, and *Kahata Wee*. The differences observed could be due to the heterogeneity of traditional rice accessions and the differences in the extraction protocol of the current study compared to that of Gunaratne et al. [1]. The TPC is known to be affected by the degree of rice milling, due to removal of phenolic compounds with the bran during rice polishing [3]. Irrespective of being pigmented or non-pigmented, many previous studies have reported lower TPC in polished rice and a higher TPC in the bran compared to brown rice [1, 3, 5]. Therefore, to maximize the benefits of the high TPC-carrying rice mentioned in the current study, it is advisable to consume them with minimal polishing.

Flavonoids are a class of secondary plant metabolites consisting of important antioxidant and chelating properties. Flavonoids such as anthocyanins, flavonols and proanthocyanidins present in rice have beneficial health effects due to their antioxidant and anti-inflammatory activities [25]. In the current study, TFC ranged from 97.70 ± 1.26–5.13 ± 0.45 mg RE/100 g, reporting a significantly higher (*P* < 0.05) mean TFC in pigmented rice accessions compared to its non-pigmented counterpart. Shen et al. [7] reported similar results, where TFC in non-pigmented white rice was 88.6–170.7 mg RE/100 g) and red pigmented rice was 108.7–190.3 mg RE/100 g. Min et al. [25] reported significantly higher (*P* < 0.05) TFC in pigmented rice compared to non-pigmented rice, and a higher TFC was reported in purple rice compared to red rice. To the best of our knowledge, TFC has not been assessed in Sri Lankan rice accessions. Therefore, the accession *Masuran* which carries a higher TFC depicts a greater potential to be used in the nutraceutical industry and/or in the food industry as a functional food.

The PAC is a class of polymeric phenolic compounds consisting of flavon-3-ol units which include catechin, epicatechin, 3-O-gallates and epigallates [11, 26]. Catechin and epicatechin are particularly abundant as proanthocyanidins in the rice bran [11]. The PAC of the studied rice accessions ranged between 57.43 ± 4.00–333.27 ± 4.00 mg CTE/100 g, with a significantly higher (*P* < 0.05) PAC in pigmented accessions compared to most non-pigmented rice accessions. These findings are comparable with Gunaratne et al. [1] which reported a PAC range of 107 ± 2.00–227 ± 5.00 mg CTE/100 g for brown rice. The undetectable levels of PAC reported in non-pigmented rice in Gunaratne et al. [1] contradicts with the present study. Nevertheless, comparable results were reported by Min et al. [26], where a PAC of 9–202 mg CTE/100 g was reported for pigmented rice accessions and a PCA of 6–7 mg CTE/100 g was reported for non-pigmented rice accessions.

According to the findings of Gunaratne et al. [1], PAC of brown rice was approximately one tenth of the PAC found in rice bran. Thereby, the bran of the investigated accessions can contain approximately ten times more PAC compared to the PAC reported in brown rice in the current study. Premakumara et al. [24] discussed the potential use of the rice bran of Sri Lankan traditional pigmented rice accessions in developing functional foods, nutraceuticals and pharmaceuticals for diabetics and those suffering from high anti-amylase and anti-glycation. Hence, Ld 368 and At 311 can be recommended as high PAC containing improved rice accessions and further investigations are warren to reap its potential applications in industry.

The RSA measures reducing-capacity of antioxidants to break-free radical chain reactions [27]. The RSA of phenolic fractions of the evaluated rice accessions ranged from 10.57 ± 0.10–91.36 ± 0.87%, however, RSA in pigmented rice accessions were significantly higher compared to non-pigmented accessions. The radical scavenging activity influences the inhibition of cholesterol oxidation [11, 28]. Therefore, the identified rice accessions with high RSA levels can be further investigated to understand their potential use as nutraceuticals and as functional food ingredients.

Clustering of rice accessions considering the antioxidant properties; TPC, TFC, PAC, and RSA resulted two distinct clusters corresponding to the pericarp colour. Among the rice accessions grouped into the Cluster 1 which represented the pigmented cluster, sub-cluster C2 (*Kahata Wee*, *Madathawalu*, *Sudu Heenati*, and Bw 272-6B) carried the highest TPC, while sub-cluster C3 (At 311 and Ld 368) carried the highest PAC. Hence, the rice accessions in the above emphasized sub-clusters would be potentially important for parental selection in breeding programs and for developing mapping populations for further investigation of their genetics. Further, according to the results of the current study, pigmented accessions; *Kahata Wee*, *Madathawalu*, *Deweraddiri*, *Masuran*, *Kalu Heenati*, *Sudu Heenati*, *Pokkali*, At 311, Bw 272-6B and Ld 368 have shown higher values with respect to all four antioxidant parameters; TPC, TFC, PAC, and RSA. Hence, these rice accessions can be recommended for further investigations to realize its potential in nutritional and nutraceutical improvement of rice grain quality.

The RSA showed a significant positive correlation with TPC, TFC and PAC. Previous findings have also reported a similar relationship between the RSA and antioxidant content, quantified as TPC, TFC, and PAC [23, 25]. In contrast to these findings, Premakumara et al. [24] reported a no significant correlation between TPC and RSA quantified with DPPH assay, however, in the same study a significant positive correlation was reported between TPC and RSA when quantified through ABTS assay.

## Defining exon 6 based *Rc* gene haplotypes and deducing its correlation with antioxidant properties

Mutations in the exon 6 of *Rc* gene affects the functionality of the bHLH protein regulating the pericarp colour in rice grains. According to Sweeney et al. [17] the 14-bp InDel at 1,408–1,421-bp position on the mRNA creates a frameshift mutation resulting a stop codon, prematurely truncating the protein ahead of the bHLH domain, leading to a white pericarp. However, in the current study, the rice accessions *Inginimitiya* and *Suwandel* carried a white pericarp even in the absence of the 14-pb InDel. Hence, the pericarp colour of these two rice accessions could not be explained solely based on the 14-bp InDel in the exon 6 of *Rc* gene. According to Sweeny et al. [15], an Adenine at the A/C SNP at 1,353-bp position in the mRNA creates a premature stop codon ahead of the bHLH domain and restore the impact created by the 1-bp InDel and/or 14-bp InDel positioned distally. Accordingly, the white pericarp of the rice accessions *Inginimitiya* and *Suwandel* could be explained as a loss of function in the bHLH transcription factor involved in the proanthocyanidin production, due to a premature stop codon created at the A/C SNP site due to carrying Adenine. At the 1-bp InDel (G/-) at 1,388-bp position in the mRNA corresponding to the exon 6 of *Rc* gene all accessions in the study panel carried a Guanine. According to Brooks et al. [18], a deletion at this site could lead to a frameshift in the resulted amino acid sequence, reverting the impact of the deletion occurring at the 14-bp InDel. As a result all three-target sites A/C SNP, 1-bp InDel and 14-bp InDel can be reported as critical for defining the pericarp colour in rice. Sweeney et al. [17] defined 11 haplotypes based on the entire *Rc* gene. However, in the current study, in light of predicting the pericarp colour of rice, three haplotypes (H1-H3) were defined based on the critical A/C SNP, 1-bp InDel and 14-bp InDel polymorphisms in the exon 6 of *Rc* gene. Of the three haplotypes, H1 resulted red pigmented rice pericarp (Fig. 5; H1: C/G/+), and H2 and H3 resulted non-pigmented rice with white pericarp (Fig. 5; H2: A/G/+ and H3: C/G/-). When the haplotypes H1-H3 were related to TPC, TFC, PAC and RSA, the mean of these parameters in H1 haplotype representing the pigmented rice accessions was significantly (*P* < 0.05) higher compared to that of H2 and H3 haplotypes representing the non-pigmented rice accessions. The significant (*P* < 0.05) differences observed in the mean antioxidant properties of the haplotypes H1-H3 (Table 2) validates the use of these haplotypes as a molecular tool for the selection of rice accessions with better antioxidant properties and using these haplotypes for the prediction of pericarp colour in grains. Hence, the haplotype-based screening introduced in the current study would be an alternative to conduct early selections for antioxidant content and pericarp colour in rice breeding programs as a substitute to currently adopted time consuming physico-chemical methods.

## Conclusions

The study revealed variation in antioxidant properties, where the majority of pigmented rice accessions exhibited higher TPC, TFC, PAC and RSA than the non-pigmented rice accessions. Compared to others, the pigmented accessions *Sudu Heenati* and *Madathawalu* reported a significantly higher TPC, and *Masuran* a significantly higher TFC. The accession Ld 368 reported the highest PAC and it was not significantly different to At 311. The rice accessions *Sudu Heenati* and *Deweraddiri* reported the highest levels of RSA. However, they were not significantly different to *Kalu Heenati*, *Madathawalu*, At 311, *Masuran*, Ld 368, Bw 272-6B, *Pokkali*, At 362, and *Wanni Dahanala* for RSA. Therefore, these rice accessions should be further investigated to explore their antioxidant properties for commercial use. Based on three-critical target sites in the exon 6 of *Rc* gene in rice (A/C SNP, 1-bp InDel, and 14-bp InDel), three haplotypes H1 (C/G/+), H2 (A/G/+) and H3 (C/G/-) were defined. A significant (*P* < 0.05) difference was reported between the mean antioxidant properties TPC, TFC, PAC and RSA of H1 haplotype with the means of H2 and H3 haplotypes. Hence, using the defined haplotypes, successful selections can be conducted to identify rice accessions/breeding lines carrying higher antioxidant properties and predict grain pericarp colour as an alternative to currently adopted physico-chemical methods.

## Electronic supplementary material

Below is the link to the electronic supplementary material.

Supplementary Material 1

## Data Availability

Sequence data generated in the current study are deposited in National Center for Biotechnology Information (NCBI) under GenBank accession numbers PP592897 - PP592928.
