# Supplementary material for: Assessment of antioxidant properties in selected pigmented and non-pigmented rice (Oryza sativa L.) germplasm and determination of its association with Rc gene haplotypes
Source: BMC Plant Biol. 2024 Sep 28;24:884. doi: 10.1186/s12870-024-05623-2 (PMC11438363; doi:10.1186/s12870-024-05623-2)
Supplement: Supplementary file 1 — Supplementary Material 1 [file 12870_2024_5623_MOESM1_ESM.docx]

**Table 1:** Pedigree, type and accession number, pigmentation category according to descriptors and age-group of selected rice accessions

| **Rice Accession** | **Pedigree** | **Type and accession number*** | **Pigmentation according to**  **varietal description** | **Age group (months)** |
| --- | --- | --- | --- | --- |
| *Deveraddiri* | - | Sri Lankan traditional rice accession (RRDI 243) | Pigmented | 4 - 4.5 |
| *Dik Wee* | - | Sri Lankan traditional rice accession (RRDI 246) | Pigmented | 4 - 4.5 |
| *Dular* | - | Indian traditional rice accession (RRDI 252) | Pigmented | 3.5 |
| *Gonabaru* | - | Sri Lankan traditional rice accession (RRDI 28) | Non-pigmented | 4 - 4.5 |
| *Herath Banda* | - | Sri Lankan traditional rice accession (RRDI 280) | Pigmented | 3.5 |
| *Hondarawalu* | - | Sri Lankan traditional rice accession (RRDI 285) | Pigmented | 3.5 |
| *Inginimitiya* | - | Sri Lankan traditional rice accession (RRDI 1234) | Non-pigmented | 4 - 4.5 |
| *Kahata Wee* | - | Sri Lankan traditional rice accession (RRDI 324) | Pigmented | 4 |
| *Kalu Heenati* | - | Sri Lankan traditional rice accession (RRDI 333) | Pigmented | 3.5 |
| *Kuruluthuda* | - | Sri Lankan traditional rice accession (RRDI 69) | Pigmented | 4 - 4.5 |
| *Madathawalu* | - | Sri Lankan traditional rice accession (RRDI 1312) | Pigmented | 3.5 |
| *Masuran* | - | Sri Lankan traditional rice accession (RRDI 86) | Pigmented | 4 - 4.5 |
| *Pachchaperumal* | - | Sri Lankan traditional rice accession (RRDI 799) | Pigmented | 3 |
| *Pokkali* | - | Indian traditional rice accession (RRDI 809) | Pigmented | 3.5 |
| *Raddel* | - | Sri Lankan traditional rice accession (RRDI 133) | Non-pigmented | 5 - 6 |
| *Sudu Heenati* | - | Sri Lankan traditional rice accession (RRDI 491) | Pigmented | 4.5 |
| *Suduru Samba* | - | Sri Lankan traditional rice accession (RRDI 903) | Non-pigmented | 3.5 |
| *Sulai* | - | Sri Lankan traditional rice accession (RRDI 502) | Pigmented | 3.5 - 4 |
| *Suwandel* | - | Sri Lankan traditional rice accession (RRDI 579) | Non-pigmented | 3.5 |
| *Wanni Dahanala* | - | Sri Lankan traditional rice accession (RRDI 591) | Pigmented | 3.5 |
| At 306 | IR 49517-41-1-6-2-3/At 405 | New improved variety developed by RRS, Ambalantota | Non-pigmented | 3 |
| At 311 | At 306/ At 3-105 | New improved variety developed by RRS, Ambalantota | Pigmented | 3 |
| At 362 | At 85-2/ Bg 380 | New improved variety developed by RRS, Ambalantota | Pigmented | 3.5 |
| At 405 | At 402/ Basmathi 442 | New improved variety developed by RRS, Ambalantota | Non-pigmented | 4 |
| Bg 352 | Bg 380/ Bg 367-4 | New improved variety developed by RRDI, Bathalagoda | Non-pigmented | 3.5 |
| Bg 358 | Bg 12- 1/ Bg 1492 | New improved variety developed by RRDI, Bathalagoda | Non-pigmented | 3.5 |
| Bg 360 | 3346/IR 36//*Senerang* | New improved variety developed by RRDI, Bathalagoda | Non-pigmented | 3.5 |
| Bg 366 | Bg 300/94-2236//Bg 300/ Bg 304 | New improved variety developed by RRDI, Bathalagoda | Non-pigmented | 3.5 |
| Bg 94–1 | IR262/ Ld 66 | New improved variety developed by RRDI, Bathalagoda | Non-pigmented | 3.5 |
| Bw 267–3 | Ld 125/ Bw 248-1 | New improved variety developed by RRDC, Bombuwela | Non-pigmented | 3.5 |
| Bw 272–6B | Bw 242-5-5/ Bw 259-3 | New improved variety developed by RRDC, Bombuwela | Pigmented | 3 |
| Ld 368 | Ld 356/ Ld 99-14-11 | New improved variety developed by RRC, Labuduwa | Pigmented | 3.5 |

^*^Accession number as numbered by Department of Agriculture, Sri Lanka.

Breeding stations abbreviated as RRDI: Rice Research and Development Institute; RRS: Rice Research Station; RRDC: Regional Rice Research and Development Centre; RRC: Rice Research Centre
